# Discordances Between Serology and Culture for Strongyloides in an Ethiopian Adopted Child With Multiple Parasitic Infections: A Case Report

**DOI:** 10.1097/MD.0000000000003040

**Published:** 2016-03-11

**Authors:** Antoni Soriano-Arandes, Elena Sulleiro, Francesc Zarzuela, Edurne Ruiz, Isabel Clavería, Mateu Espasa

**Affiliations:** From the Pediatric Infectious Diseases and Immunodeficiencies Unit, Unit of International Health-Tuberculosis Drassanes-Vall Hebron, Hospital Universitari Vall d’Hebron (AS-A); Unit of International Health Drassanes-Vall Hebron, Programa Especial de Malalties Infeccioses (PROSICS), Hospital Universitari Vall d’Hebron (FZ, ER, IC); and Department of Microbiology, Hospital Universitari Vall Hebron, Barcelona, Spain (ES, ME).

## Abstract

Rationale: infectious diseases screening of international adoptees is complex because of the concurrence of different pathogens in a child at same time. We describe an international adopted child born at Ethiopia infected by 5 different pathogens (*Hymenolepis nana*, *Giardia intestinalis*, *Entamoeba histolytica*, *Strongyloides stercoralis*, and *Trichuris trichiura*), 2 of them *S. stercoralis* and *E. histolytica* with a capacity to develop severe clinical complications if not detected promptly with appropriate diagnosis tests.

Concerns of the patient: according to the screening protocol a stool sample is always processed for culture addressed to find out protozoan and helminthic pathogens but not specifically for *S. stercoralis*. Only, when eosinophilia is detected 3 serial stool samples are collected to rule out intestinal parasitic infection including *S. stercoralis*.

Interventions: in our case, *S. stercoralis* would not have been detected if we had followed the protocol because eosinophilia was absent and its specific serology was negative. Fortunately, the initial inclusion of the feces charcoal culture for *S. stercoralis* allowed us to detect this infection.

Outcomes: discordances between direct methods such as culture and indirect as serology or antigen test forces us to be very cautious before ruling out *S. stercoralis* or *E. histolytica* infection, respectively. Also, if a child from tropical areas has persistent symptoms (such as diarrhea or fever) that have not been treated we have to rule out other infections that have not been detected yet.

Main lessons: The introduction of different sequencing tests and the insistence to find out pathogens such as *S. stercoralis* or *E. histolytica* was determinant to be able to cure this symptomatic child and to prevent potential severe clinical forms in case of immunosuppression.

## INTRODUCTION

The screening of infectious diseases of international adoptees (IAs) is complex because of the concurrence of different pathogens in a child at same time. In fact, multiparasitism in children is common. Where possible, IAs should be evaluated at a clinic or a center specializing in international adoption, as specialized expertise and a multidisciplinary approach are often required for optimal evaluation and care of these children.^1,2^ Infections for which IAs are at higher risk and therefore require screening including viral hepatitis A (HAV), B (HBV), and C (HCV) virus, human immunodeficiency virus (HIV), bacterial (syphilis and tuberculosis), and parasitic infections (stool helminths and protozoa). When the child has eosinophilia search for helminth infection is essential to obtain the diagnosis. Also, in cases with persistent eosinophilia, tests for *Toxocara canis*, *Strongyloides*, and for *Schistosoma* are mandatory.^3^

A great number of IAs has been observed in the last 20 years in Spain; however, the overall global trend in last decade is decreasing. According to the Ministry of Health, the number of IAs has declined between 2008 (3156 cases) and 2012 (1669). Ethiopia, in recent years, has been the 3rd most common (after Russia and China), although in 2008 was the 2nd most common, country of origin of IAs.^4^ Also, Ethiopia is 1 of the 3 poorest countries in the world: their income per capita is $1110, life expectancy is 62 to 65 years and the under 5-year mortality rate is 68 per 1000 live births. It has serious deficiencies in health care and endemic droughts occur in many regions, cyclically causing famines.^5^

Our aim is to show a relevant case of an IA child coming from Ethiopia with 5 different parasitic infections from which 2 of them can have severe consequences if are not detected promptly with appropriate diagnosis tests. Moreover, we detected a discrepancy between serological/antigen tests and culture results for *Strongyloides stercoralis* and *Entamoeba histolytica* infection.

### Case Report

A 21-month-old toddler was visited at the Unit of International Health Drassanes-Vall Hebron (Barcelona, Spain), on September 5th of 2013, to do an initial health screening. He was adopted from Ethiopia and arrived on July 17th of 2013 in Spain. Reviewing the original Ethiopian document for international adoption he had no known allergies, no blood transfusions, and no previous diseases. Immunization registry data were completed following Ethiopian Vaccination Health Program. Following the CARE guidelines (http://www.care-statement.org/) we constructed a timeline table to provide a framework for a better comprehension of the follow-up of this case report (Table 1).

**TABLE 1:**
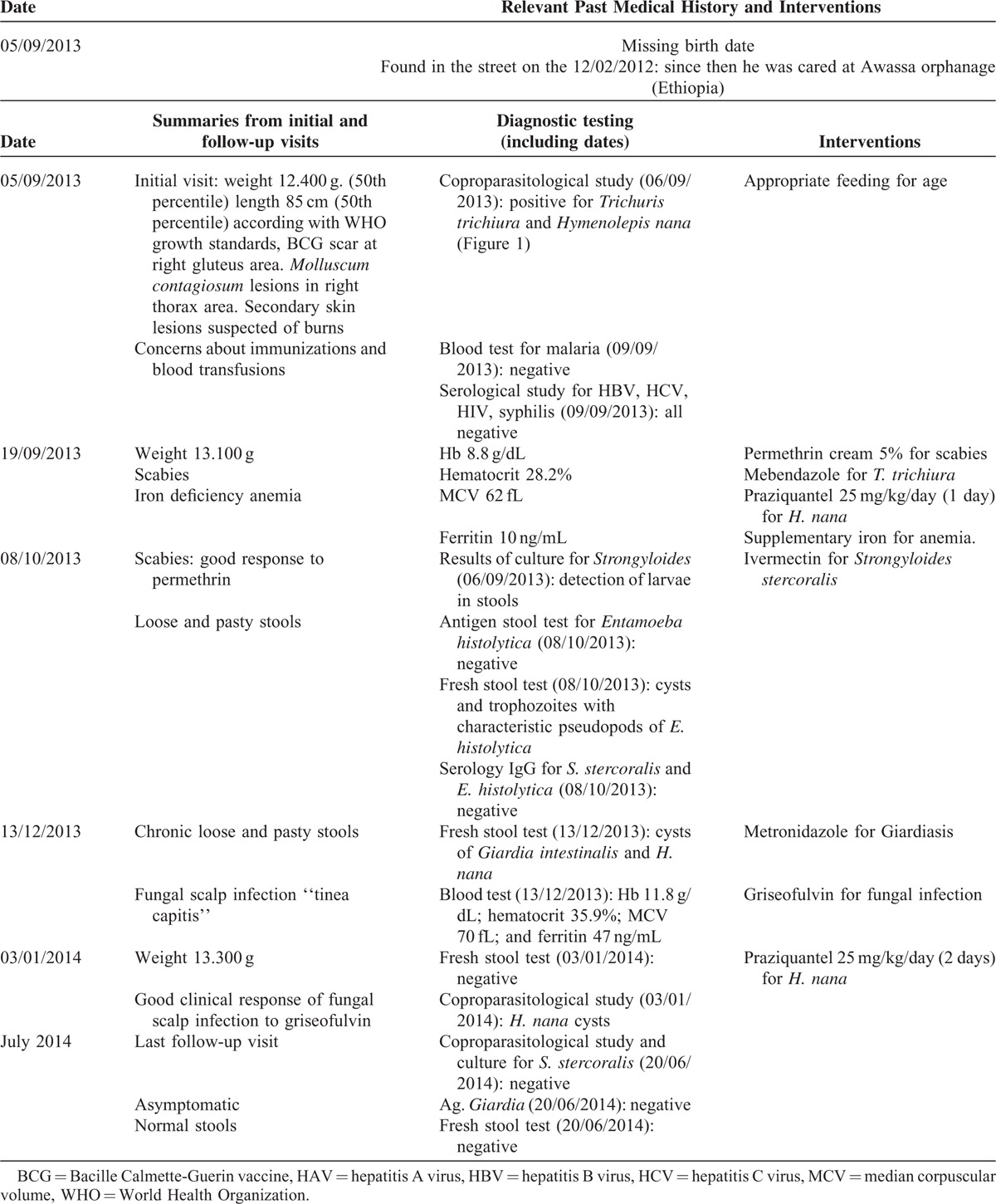
Timeline of the Following Up Course for the Ethiopian Adopted Child

On physical examination weight (12.4 kg) and height (84 cm) were on percentile 50 according to the World Health Organization pediatric growth charts. Bacille Calmette-Guerin scar was found in right gluteus area. A diverse number of skin lesions were observed including *Molluscum contagiosum* on the right thorax surface, burn rounded lesions along the body surface, and scabies lesions affecting all the fingers of hands and feet. No other clinical findings of interest were observed.

**FIGURE 1:**
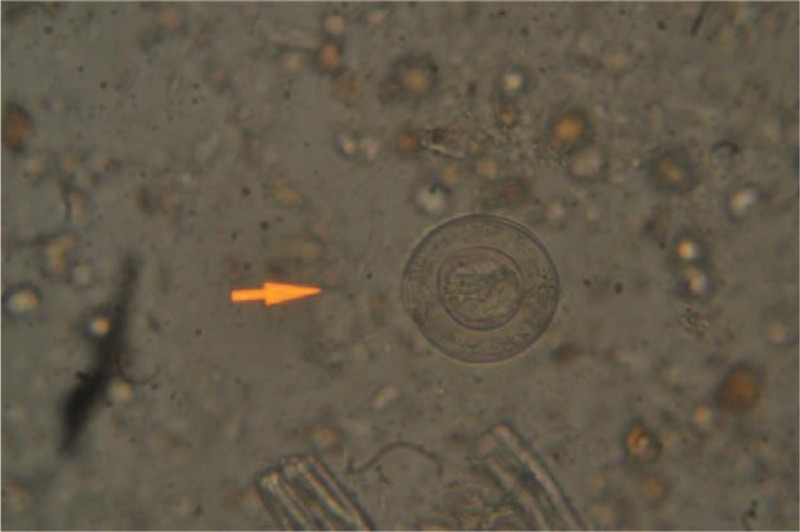
*Hymenolepis nana* cysts observed on the fresh stool exam of the child.

**FIGURE 2:**
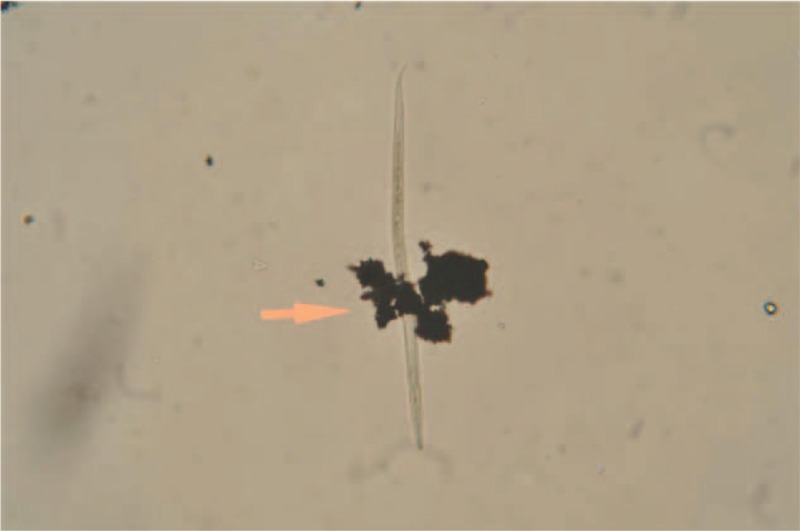
*Strongyloides stercoralis* larvae found in the charcoal culture of the stool sample of the child.

**FIGURE 3:**
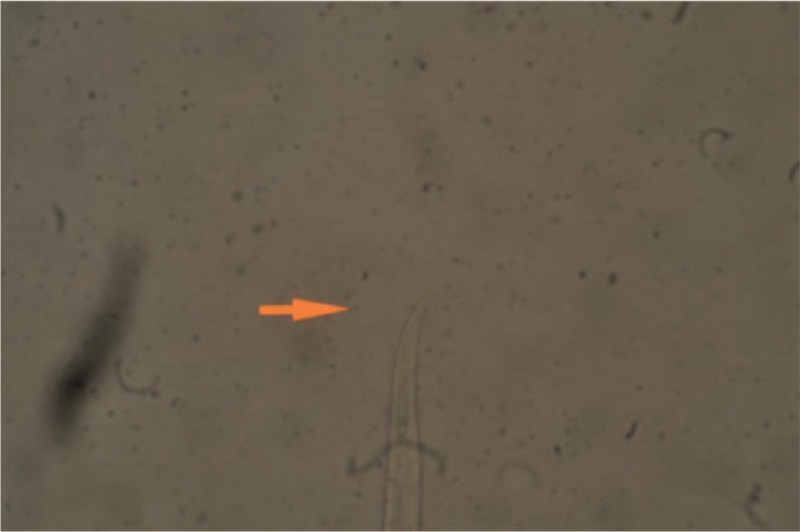
A detailed image of the tail of a *Strongyloides stercoralis* larva.

Laboratory evaluation showed anemia; hemoglobin 8.8 g/dL, hematocrit 28%, no eosinophilia (472 cells/mm^3^), and median corpuscular volume 62 fL; decreased iron tissue levels (ferritin 10 ng/mL), and normal liver and renal function. No hemoglobin-related disease was detected. Serology for *Treponema pallidum*, HIV, HBV, and HCV were negative; and positive for HAV and Varicella Zoster virus. Other infectious diseases screening tests included: negative malaria blood smear, negative tuberculin skin test, but the parasitological stool examination showed cysts of *Hymenolepis nana* (Figure 1), eggs of *Trichuris trichiura*, and cysts of *Entamoeba* spp. Charcoal culture for *S. stercoralis* detected larvae forms in stools (Figures 2 and 3). The clinical evolution and the follow-up visits are described in Table 1. Treatment with permethrin cream 5% was given for scabies, mebendazole for *T. trichiura*, praziquantel (2 days) for *H. nana*, supplementary iron for anemia, ivermectin for *S. stercoralis*, metronidazole for Giardiasis, and griseofulvin for fungal infection.

Ethical approval was waived because we did not modify our clinical care to the patient due to any research study. Informed consent to publish the case was obtained by the mother's patient.

## DISCUSSION

Screening for infectious or communicable diseases is commonly done when an IA arrives due to their susceptibility to develop and carry acute or chronic infectious diseases.^2^ Integrated protocol including screening for HAV and HBV,^6,7^ HIV infection, syphilis, and latent TB infection is applied in most of the countries. So, majority of studies on IAs are mainly addressed to know their serological status of preventable infectious diseases^6–11^ and to determine their vaccination immunological status.^11^ On the other hand, some studies are focused in the risk of infectious diseases transmission from IAs to their adoptive families.^12–14^ As an example, a measles outbreak was recently reported in United States (US) from 3 Chinese IAs with cerebral palsy because the China pharmacopeia vaccine includes encephalopathy as a contraindication for vaccination. The authors concluded that US should reconsider allowing vaccination exemptions for IAs unless there are true medical contraindications to vaccination, and US pediatricians should counsel adopting parents to ensure that their child is up-to-date on recommended vaccinations before coming to the US.^15^

Intestinal parasites infections are very common in children who have been living in Sub-Saharan Africa, sometimes with coinfections by different parasites, as it has been showed previously.^16^ According to this issue, our case demonstrated the presence of 5 parasite pathogens in an IA coming from Ethiopia. Some of them, *T. trichiura* and *H. nana* are not able to invade intestinal mucosa and its severity or chronicity is limited. However, other pathogens detected in this child have a capacity to affect other organs, such liver in the case of *E. histolytica* or a hyper infection by *S. stercolaris* in case of immunosuppressive status. Prevalence of *S. stercoralis* in Ethiopia has been studied in HIV-infected patients,^17^ in patients with diarrhea,^18^ in general population,^19^ in children,^20^ and in Ethiopian immigrants^21^ showing values between 0.4% and 5.9%. Since these parasitic infections are commonly asymptomatic, all IAs should be screened for parasite infections with a potential capacity to produce severe diseases, as showed in the study by Hénaff et al,^2^ where 8% of the cases presented with severe infections.

According to our screening protocol a single stool sample is always culture for protozoan and helminthic pathogens but not specifically for *S. stercoralis*. Only, when eosinophilia is detected 3 serial stool samples are collected to rule out intestinal parasitic infection including *S. stercoralis*. In our case, *S. stercoralis* would not have been detected if we had followed the protocol because eosinophilia was absent and its specific serology was negative. Fortunately, the initial inclusion of the feces charcoal culture for *S. Stercoralis*, due to a mistake into the procedure of the request to the laboratory, allowed us to detect this infection. This type of culture is cheap, easy to be done, and more sensitive than concentrated stool microscopy examination. It also allows for easier speciation between *Strongyloides* and hookworm due to development of rhabditiform larvae of hookworm. Charcoal is used to maintain pH and to provide a medium in which the larvae can develop, and allow larvae to develop to the filariform stage to further aid in diagnosis.^22^

Serology for *Strongyloides* is a useful tool but it might overestimate the prevalence of disease due to cross-reactivity with other nematode infections and its difficulty distinguishing recent from past (and cured) infections.^23^ Also, as described in our case a negative serology (false negative) cannot exclude the infection completely because sensitivity of different serological tests is between 56% and 100%.^23^ Recently, a summary of different approaches to *S. stercoralis* diagnosis, including molecular biology techniques, showed serology as the most sensitive test with a negative predictive value of 100% at low prevalence settings.^24^ However, this is not so for a recently acquired infection as suspected in this case report. Therefore, in cases of clinical suspicion, the clinician should be reminded that the serology is not sufficient to rule out the infection if it gives a negative result.^24^ To evaluate treatment efficacy is still a major concern because direct parasitological methods might overestimate it and the serology has not yet been well evaluated; even if there is a decline in antibody titers after treatment, it is slow and needs 6 to 12 months after treatment which can cause a substantial loss to follow-up in a clinical trial.^23^

Secondly, *E. histolytica* is a protozoan pathogen that comprises 2 genetically distinct but morphologically indistinguishable species. *E. histolytica* can cause invasive intestinal and extra intestinal disease, while *Entamoeba dispar* cannot. Identification and differentiation of *E. dispar* and *E. histolytica* in stool sample by microscopy is most of times not possible. Microscopy has low sensitivity and high specificity, low negative predictive value and high positive predictive value in comparison with enzyme-linked immuno sorbent assay. *E. histolytica* antigen detection enzyme-linked immuno sorbent assay tests could identify the pathogenic *Entamoeba* and easy to perform. It does not require experienced microscopists and can therefore be recommended for stool's screening worldwide, and the results could be taken to address treatment.^25^ The use of all methods in combination and evaluation together with the clinical symptoms seems to be the best approaches for the laboratory diagnosis of patients with suspected Amebiasis.^26^

## CONCLUSION

Adopted children from tropical areas with persistent symptoms (such as diarrhea or fever) should be screened for other infections that have not been detected yet. The introduction of different sequencing tests and the insistence to find out these pathogens is determinant to prevent potential severe clinical forms. Negative serology for *S. stercoralis* cannot exclude the infection completely, and discordances between fecal culture and serology need to be further investigated in prospective studies on international adopted children.
